# COVID-19 mothers’ mother–baby bonding, feeding practices, postnatal care experiences in Qatar: A mixed-methods approach

**DOI:** 10.18332/ejm/209553

**Published:** 2025-08-31

**Authors:** Laura C. Moya Falcon, John Paul Ben T. Silang, Safa El-Arwa Hadid, Khadije Bargaoui, Mariama Lilei Kassay, Jussara Da Silva Brito, Nesiya Hassan, Albara Mohammad Ali Alomari, Teresa Sandra Erice Rivero, Kalpana Singh

**Affiliations:** 1 Women’s Wellness and Research Center, Hamad Medical Corporation, Doha, Qatar; 2 Asiha Bint Hamad Al-Attiyah Hospital, Hamad Medical Corporation, Doha, Qatar; 3 Al- Wakrah Hospital, Hamad Medical Corporation, Al Wakrah, Qatar; 4 Nursing and Midwifery Research Department, Hamad Medical Corporation, Doha, Qatar; 5 University of Doha for Science and Technology, Doha, Qatar; 6 The Cuban Hospital, Hamad Medical Corporation, Dukhan, Qatar

**Keywords:** breastfeeding, postpartum, COVID-19, pandemic, postnatal care, impaired bonding

## Abstract

**INTRODUCTION:**

The study aim was to examine mother–infant bonding, feeding practices, and postnatal care experiences of mothers diagnosed with COVID-19 in hospital settings from 2020 to 2022.

**METHODS:**

A mixed-methods research design was conducted, involving 117 participants in a cross-sectional online survey and 11 phone interviews. The study was conducted among mothers diagnosed with COVID-19 by PCR test and admitted to four maternity facilities in Qatar from 1 May 2020 to 16 January 2022. The Postnatal Bonding Questionnaire was used to examine mother-baby bonding, and interviews were conducted to gain a deeper understanding of the overall postnatal experience. Descriptive statistics, t-tests, and ANOVA were applied to analyze associations between postnatal bonding scores and various factors.

**RESULTS:**

Participants had a postnatal bonding mean score of ≥12, which indicated impaired bonding (mean=12.0, SD=4.7). Mode of birth and postnatal bonding scores were correlated, especially those with instrumental deliveries (mean=30.2, SD=12.0, p<0.001). Five themes emerged illustrating the respondents' adaptive strategies and the build-up of impaired bonding during the pandemic. These themes underscored the need for support in enhancing mothers' coping and resilience to the challenges during the pandemic.

**CONCLUSIONS:**

The pandemic has significantly impacted maternal–infant bonding, as evidenced by increased reports of maternal stress, reduced physical contact, and limitations on partner support. Midwives and other healthcare professionals play a pivotal role in supporting, educating, and reassuring women about providing safe, high-quality care during the crisis. Further research is essential to develop evidence-based guidelines and to examine the long-term consequences of disrupted bonding on infant development.

## INTRODUCTION

Maternal–infant bonding is a crucial psychological process involving the reciprocal emotional connections between mothers and their infants^1^. The connection starts during pregnancy but intensifies during the postpartum period as women undergo many physical and emotional transformations^2^. This natural bonding enables mothers to provide optimal care for their newborns despite facing challenges during puerperium^3^. The quality of the bonding is impacted when mothers find it challenging to feel affectionate toward their babies. This condition is termed impaired bonding^4^. Several studies linked impaired bonding to maternal stress, postpartum depression, and lack of social support^5^. Bonding has also been linked to the behavioral development of children, including adjustments like establishing emotional connections^6^. Impaired bonding can arise through many environmental, physiological, and psychological influences and the COVID-19 pandemic was no exception. In fact, maternal-infant bonding was one of the most common concerns in postnatal care during the COVID-19 pandemic. Other than impaired bonding, added concerns are uncertainties and probable changes in breastfeeding and clinical care protocols during postpartum period. Postnatal bonding is accentuated with breast feeding and positive postnatal care experience. In breastfeeding, the intimate practice of physical closeness and skin contact initiates profoundly the emotional bond between the mother and the baby^7^. On the other hand, several studies also suggested that the quality of care facilitates mother–baby bonding. A study showed that with caring, the mother bonds with the baby and establishes support from the care provided, which enhances mother–baby bonding^8^.

At the beginning of the pandemic, there were no evidence-based recommendations about how to care for COVID-19-positive pregnant women and their newborn babies. There were limited sources about guidelines for the neonatal care of babies of women who tested positive in the third trimester^9^. Studies suggested a potential risk for droplet transmission from confirmed COVID-19-positive mothers or caregivers to neonates. However, transmission was deemed unlikely if proper hygiene precautions were undertaken following effective parental education^10^. The uncertainties of the risks and consequences of infections and the changes in the surroundings may increase maternal stress^11^. These changes in perinatal care were implemented in healthcare facilities to reduce the transmission of COVID-19 during the early stage of the pandemic. Babies were seen as vulnerable populations, and healthcare organizations implemented extra care to mitigate the risk of COVID-19 transmission from their mothers and healthcare providers. The perinatal care for mothers was also reformed as adaptations to changes in healthcare systems and protocols were strictly necessary. In hospital settings, the changes in perinatal care protocols during the initial phase such as limiting gathering size, visitations, laboratory tests, and strict surveillance, have probably affected mother–baby interaction, breastfeeding practices, and delivery of postnatal care^2,5^. The evolving clinical guidelines may have compromised maternal behavior and affect opportunities for mother and baby to interact, which has been reportedly low in several countries during the COVID-19 pandemic^2,5,12^. However, contrasting findings were also reported that there was a positive impact of the pandemic restrictions during pregnancy and postnatal period. Women have more time for resting, mother–baby bonding, and breastfeeding due to the lockdown and restrictions, which are common among women in communities that are either confirmed or not infected with COVID-19^13^.

In Qatar, maternal and neonatal well-being are among Qatar’s national health priorities. The country has formulated strategies to sustain quality and safe healthcare services for mothers and babies, especially during the pandemic^14^. The most recent guidelines in COVID-19 recommend women to hold the baby and perform breastfeeding following proper hygiene and preventive measures. Due to the limited data, it was recommended that precautionary measures should be followed as pregnant women may easily get infected by respiratory infections due to hormonal changes during pregnancy. Two studies highlighted insights about the perinatal experience of mothers in antenatal clinics and inpatient maternity units in Qatar during the COVID-19 pandemic. A cross-sectional survey among women in third trimester period showed that fear of their newborns is associated with intent not to breastfeed. The study articulated that integrating obsessive thoughts of infection risks and compulsive hygienic practices becomes a strategic response to prevent newborn COVID-19 infection^15^. The other study was conducted during the peak of the COVID-19 pandemic, which detected anxiety and symptoms of depression among both pregnant and postnatal women due to social restrictions and worries about family safety, including their children^16^. To date, no study has examined the experience of mothers who were specifically only among confirmed cases of COVID-19. Furthermore, no studies have examined maternal infant bonding during the pandemic. Mothers may have been concerned about their babies, their own, and their family’s well-being. Access to more contextualized and evidence-based information and support in managing perinatal care is crucial. Emotional connection, bonding, and family closeness should be a priority in healthcare provision aside from protecting people from the virus. With the available evidence regarding mothers and babies’ prevention of transmission, there has been a gap in how these guidelines have impacted the perinatal period among women and the care providers in this region. So, this investigation performed a comprehensive exploration to enhance the breadth and depth of understanding experience of mothers diagnosed with COVID-19 specifically in mother– baby bonding, breastfeeding, and postnatal care experience in Qatar through a mixed-methods study.

## METHODS

### Study design

A quantitively driven sequential mixed-methods design was chosen to gain comprehensive understanding postnatal bonding during the COVID-19 pandemic. This design included quantitative data collection and analysis in Phase I, followed by qualitative data collection and analysis to explain the salient findings in Phase I. A cross-sectional research design, done via a phone survey, was conducted to assess postnatal bonding in Phase I. The variables examined include the respondents profile defined as both demographic and obstetric characteristics, and the postnatal bonding which is measured using a standardized tool indicating either presence or absence of impaired bonding. In Phase II, series of phone interviews were conducted to examine their postnatal experience of the mothers diagnosed with COVID-19 specifically in bonding, feeding practices and postnatal care.

### Sample size and sampling methods

A chart review from 1 May 2020, and 16 January 2022, was conducted to obtain a list of eligible mothers for the survey and interviews. The eligibility criteria include the following: 1) mothers who were diagnosed with COVID-19 by PCR test; and 2) admitted in the inpatient units of the selected four maternity facilities in Qatar for childbirth and postnatal care. In Phase I, to determine the appropriate sample size for a finite population of 300 women, we used a hypothesized outcome frequency of 50%, a ±5% margin of error, and a 95% confidence level. Based on these parameters, a minimum of 111 participants were required for the study. Ultimately, responses were received from 117 women for the quantitative survey. In Phase II, based on previous studies which are closely relevant to the present study, a sample of 10 of 15 is enough to achieve saturation. In our study, saturation was achieved with the 11 participants, whereby no new insights and themes emerged from the interviews.

### Instruments and data collection procedures

In Phase I, a questionnaire was used which comprised two parts. The first part of the survey examined the participants’ profiles which are defined as demographic and obstetric data. Education, employment, and delivery were defined according to degree or classification as categorical variables. The second part assessed maternal–infant bonding using the Postnatal Bonding Questionnaire (PBQ). The PBQ was developed by Brockington et al.^1^ and was reportedly valid and reliable. The tool comprises 25 questions on a 5-point Likert scale where 5 indicates ‘always’ and zero ‘never’. Postnatal bonding is defined according to the cutoff scores using PBQ in four dimensions such as impaired bonding as ≥12 with 12 items, rejection and pathological anger as ≥17 with 7 items, infant-focused anxiety as ≥10 with 4 items, and incipient abuse as ≥3 with 2 items^1^. To involve eligible Arabic-speaking women, a translated version of PBQ was used, which was validated with five content experts following the recommendations of Polit and Beck^17^ and was tested for internal consistency with 30 mothers, which showed acceptable reliability (Cronbach’s α=0.71). In this phase, convenient sampling was used whereby women who are eligible were contacted through their mobile or contact numbers registered in their profile and the survey link was forwarded accordingly. The link contains an information sheet which explains the details of the project and the survey tools.

In Phase II, an interview guide was used with primary and supplementary questions for the interviews to gain deeper understanding of the participants’ postnatal experience which includes postnatal bonding, feeding practices and postnatal care. Purposive sampling was used in selecting the participants using the same eligibility criteria as in Phase I. Women were recruited using their phone or contact numbers, and those who decided to join received copies of the informed consent after thorough discussion about the project details and their role as participants. For those who were undecided, a two-week waiting period was implemented, and they were re-contacted for their final decision. Copies of the signed informed consent were received online and were kept on the desktop of the principal investigator. Fifteen were recruited but the interviews were completed with only 11 participants as data saturation was achieved.

### Ethical considerations

Ethics approval was obtained from the Institutional Reviewed Board of Hamad Medical Corporation (MRC-01-22-040).

### Data analysis

Descriptive statistics, including frequencies and percentages, and means and standard deviations, were used to summarize sociodemographic, occupational, and health-related characteristics of the participants. The distribution of continuous variables was assessed for normality using the Shapiro-Wilk test and visual methods such as Q-Q plots. To check associations, independent t-test and one-way ANOVA were used for comparing means across the postnatal bonding score, to identify significant differences. Pearson correlation was used to examine relationships between continuous variables. Statistical significance was set at p<0.05, and all analyses were conducted using STATA 17.0.

Two experts checked the transcripts against the audio recordings in qualitative research to ensure accuracy. Thematic analysis was performed following the recommended methods of Braun and Clarke^18^. The analysis starts with familiarization of the data by reading and re-reading the transcripts. Patterns were observed and initial codes were derived and then the final codes. The codes were clustered and reviewed based on the transcripts and aim of the study. The clusters were assigned for themes which are followed by defining and naming themes. The strategy of having two experts independently analyzing the data ensures the validity, specifically dependability and authenticity of the findings. In addition, member-checking was done whereby results were shared with the participants for feedback to ensure that the interpretations align with their experience and intended meanings. The point of integration was done using qualitative findings to illustrate the underlying process of the quantitative results^19^.

## RESULTS

### Profile of the mothers

As shown in Table 1, in relation to the demographic profile, the mean age of the 117 respondents surveyed was 33 years. The majority had permanent jobs and completed a Bachelor’s degree. In relation to obstetric profile, the majority experienced vaginal delivery, others had either a cesarean section (CS) or instrumental delivery. It was noticeable that the CS deliveries increased during the pandemic which were previously reported as gradually increasing from 16.3% in 1998 to 29.8% in 2013 in Qatar during the pre-COVID period^16^.

**Table 1:** Profile of the participants from the survey in Phase I (N=117)

*Characteristics*	*n*	*%*
**Education level**		
Primary	12	10.3
Secondary	35	29.9
Bachelor’s	51	43.6
Postgraduate	19	16.2
**Employment**		
Permanent	90	76.9
Contractual	27	23.1
**Mode of delivery**		
Normal vaginal delivery	63	53.8
Cesarean	49	41.9
Other	5	4.3

### Postnatal bonding

Table 2 shows the overall postpartum bonding scores and the four subscales in postnatal bonding. Results showed a mean score of 12.0 ± 4.7 which falls within the cutoff score of ≥12.0, indicating impaired bonding. Scores for pathological rejection, infant anxiety, and incipient abuse are lower than the cutoff values indicating that these are not consistent experiences among the participants in the survey.

**Table 2:** Postnatal bonding scores of mothers in the survey in Phase I (N=117)

*Components*	*Mean (SD)*	*Median (IQR)*
Impaired bonding	12.0 (4.7)	11.0 (8.0–14.0)
Rejection pathological	6.7 (2.8)	6.0 (5.0–8.0)
Infant anxiety	2.1 (2.0)	2.0 (0.0–2.0)
Incipient abuse	0.2 (0.7)	0.0 (0.0–0.0)
Overall postpartum bonding	19.3 (8.4)	16.0 (14.0–24.0)

IQR: interquartile range.

### Association between postnatal bonding and mother’s profile

Table 3 shows the variations in postpartum bonding measures across different variables. Education level appears to influence certain bonding aspects, as postgraduate mothers recorded higher mean scores in impaired bonding (13.7 ± 4.7) and rejection pathology (7.9 ± 3.2) compared to mothers with lower levels of education, with rejection pathology showing statistical significance (p=0.038). Employment type (permanent or contractual) did not show significant differences across bonding measures. However, the mode of delivery significantly affected bonding scores, with mothers undergoing ‘other’ modes of delivery (e.g. assisted deliveries) recording the highest mean scores in overall postpartum bonding (30.2 ± 12.0, p<0.001) and rejection pathology (9.8 ± 3.5, p=0.028). Age did not significantly impact bonding metrics. These findings suggest that education level and mode of delivery may play roles in postpartum bonding experiences, warranting targeted interventions to support mothers at risk of impaired bonding (Table 3). Higher scores on the Postpartum Bonding Questionnaire (PBQ) typically indicate greater impairment in bonding rather than better outcomes. Both a higher level of education and assisted deliveries (categorized as ‘Other’) are associated with higher overall bonding scores, suggesting more difficulties in bonding. This implies that individuals with higher level of education and those who underwent assisted deliveries may be experiencing more challenges in establishing maternal–infant bonding.

**Table 3:** Correlation between profile and postnatal bonding scores in Phase I

*Characteristics*	*Impaired bonding*	*Rejection pathological*	*Infant anxiety*	*Incipient abuse*	*Overall postpartum bonding*
	*Mean ± SD*	*Mean ± SD*	*Mean ± SD*	*Mean ± SD*	*Mean ± SD*
**Education level**					
Primary	12.4 ± 5.3	5.7 ± 2.2	0.9 ± 1.2	0.0 ± 0.0	16.8 ± 4.3
Secondary	10.9 ± 3.7	6.0 ± 2.3	1.9 ± 1.7	0.1 ± 0.4	17.2 ± 6.2
Bachelor’s	11.9 ± 5.2	7.1 ± 3.0	2.5 ± 2.0	0.3 ± 0.9	20.5 ± 9.6
Postgraduate	13.7 ± 4.7	7.9 ± 3.2	2.4 ± 2.8	0.3 ± 0.7	21.8 ± 9.4
p	0.22	0.038*	0.090	0.30	0.11
**Employment**					
Permanent	11.9 ± 4.8	6.8 ± 2.8	2.3 ± 2.1	0.2 ± 0.7	19.6 ± 8.6
Contractual	12.3 ± 4.8	6.5 ± 2.9	1.6 ± 1.6	0.2 ± 0.5	18.3 ± 7.7
p	0.71	0.58	0.11	0.86	0.47
**Mode of delivery**					
Normal vaginal delivery	12.7 ± 4.9	6.8 ± 2.9	2.2 ± 2.0	0.3 ± 0.9	20.4 ± 8.7
Cesarean	10.4 ± 3.6	6.3 ± 2.4	1.9 ± 1.8	0.0 ± 0.2	16.9 ± 6.3
Other	18.2 ± 6.6	9.8 ± 3.5	3.6 ± 3.8	0.6 ± 0.9	30.2 ± 12.0
p	<0.001*	0.028*	0.18	0.052	<0.001*
**Age** (years)	0.0272	0.0952	0.0493	0.0997	0.0599
p	0.7712	0.3074	0.5978	0.2846	0.521

* p<0.05 significant.

### Themes

Eleven mothers previously diagnosed with the COVID-19 pandemic were interviewed about their postnatal experience, including breastfeeding and postnatal bonding. Two were Arab and ten were non-Arab women. Three were primigravid, six had CS birth, and the majority initiated breastfeeding after discharge. Based on the participants’ narratives, five themes described women’s experiences during the postnatal period with the new normal during the pandemic. Regarding qualitative findings, narratives describing impaired bonding were explored (Table 4).

**Table 4:** Thematic descriptions about postnatal experience during COVID-19 from eleven mothers in Phase II

*Theme*	*Subtheme*	*Narratives*
**Navigating fear in uncertainty**	Uncertainties due to limited information Fear of the baby’s condition Fear of death	P2: ‘*We don’t know what will happen after I got COVID-19. I was also afraid I may die* … *I was afraid I had COVID and maybe my baby will have COVID also.’* P4: *‘So, this was new*… *didn’t know how this would affect my baby and we’re hearing about people dying and that would affect my baby.’*
**Experiencing separation and its consequences**	Physical separation Limited postnatal bonding Emotional distress Less breastfeeding opportunities	P1: *‘I’m missing out* … *cuddling the baby*… *kissing the baby. In a way there was as sort of emotional distance because I didn’t get to do those things.’* P3: *‘After the delivery, they did not show me my baby immediately*… *I came back in the room, and I really wanted to see my baby because I’ve been praying for my baby a lot.’* P4: *‘I don’t really touch her much* … *I refused breastfeeding* … *I don’t want her to have a contact.’* P6: *‘After the breastfeeding, the baby was separated from me. This also made me feel disturbed.’*
**Role of support in maternal well-being**	Support from healthcare providers Limited family support Lack of support affects women’s emotions	P1: *‘With other deliveries, I have a lot of support. We were well prepared like my husband.* *But for this, I was all be myself.’* P3: *‘I was really happy with the care and the support that I got from* … *our doctors to nurses to caretakers, everyone.’* P8: *‘I was all alone* … *my husband and my mum were not there. I saw the baby after hours* … *but the staff did the best to make me comfortable. It was a sad experience.’*
**Adapting with coping strategies**	Spiritual and emotional coping Distance resolved with virtual platforms Practical decision-making for self	P2: *‘I was praying and talking to my baby like that to calm myself.’* P3: *‘We had video calls* … *we felt like love was calling from distance.’* P4: *‘I tried to suppress my emotion, for the sake, you need to be strong and everything.’* P10*:‘Good hygiene and proper decisions. I have to manage the situation and time well. Panicking will not help.’*
**Acquiring resilience**	Acceptance to new ways Positive response to challenges Reconnection brings joy Viewed pandemic as a learning experience	P4: *‘I mean the happiness when you reconnect with your baby* … *the overwhelming experience is doubled because last time you weren’t able to hold her.’* P6: *‘I had a frame of reference* [previous pregnancies] *so I knew some things before, the experience now is a lot more different.’* P8: *‘I started communicating to others especially in the workplace. A lot of improvement, I can manage the baby when I am alone.’* P8: *‘I just had to get on with it* … *I couldn’t change what was happening, but I could react differently.’*

*Theme 1: Navigating fear in uncertainty*

The interplay of mortality concerns, mother’s protective instincts, and uncertainties of knowledge of COVID-19, impacts the fear of mothers during the perinatal period. The fear was brought by the uncertainties of the mother about what would happen to her and her baby. To the mother, fear of death was articulated, especially with Participant 2 (P2), which can be attributed to the reports about the infection rates and mortality due to COVID-19 in media platforms. Regarding the baby, the women’s sense of protectiveness for their newborns drove them to minimize normal bonding behaviors because of fear that they may infect the baby.

*Theme 2: Experiencing separation and its consequences*

The separation experience of the women started during the prenatal period from their families and continued postpartum from their babies. The mothers experienced both physical and emotional separation from their newborns during the pandemic. As articulated by P3, social distancing created a physical separation after birth, which negatively affected their emotions. This experience was similar to that of P1, whereby the impaired initial bonding fostered emotional detachment between the mother and her baby. P6 articulated that the separation causes distress. Triggered by fear, mothers were concerned that they might infect their babies, which affected the early initiation of breastfeeding. The mandatory face masks posed a challenge with breastfeeding in the hospital as well. Both experiences were attributed to the delay in initial bonding. Also, several mothers shared that they only started breastfeeding their children until after hospitalization or at their homes, mostly 2 weeks after their childbirth. Thus, the separation caused by social distancing and clinical guidelines disrupted opportunities for early bonding, interrupting emotional attachment. Moreover, physical separation delayed skin-to-skin contact and breastfeeding initiation.

*Theme 3: Role of support in maternal well-being*

With limited access to their families, the mothers received support from the most available resources at this stage. Both P1 and P8 articulated that they were not with their husbands and families, so they had a different experience of childbirth than before. Mostly, they felt alone, as if they were in isolation due to the risks, and it was a different experience than before when they had much support in their previous pregnancies. Nevertheless, the participants felt the support initiated by the healthcare providers, especially the nurses and midwives who stayed with them longer than others. The initial feelings of fear and emotional distress due to separation were relieved with readily available access to healthcare provider’s support. Participants 3 and 8 described joy and comfort as care providers provided support through communication, interventions, and making themselves available with personal protective equipment. Despite the lack of such resources, healthcare providers have addressed the family support system gap, enhanced maternal well-being and provided practical postnatal bonding experience.

*Theme 4: Adapting coping strategies*

Aside from the healthcare provider’s support, the participants reported several adaptive strategies that helped them cope during the pandemic. Participant 2 articulated praying, which reflects coping with spiritual practices and accepting their situation. With digital devices, women could access virtual communication with their family members. Participant 3 expressed relief at seeing photos of her newborn baby that were shared with her during the postnatal period. Another strategy was making decisions for themselves and becoming self-reliant. They adhered to clinical guidelines that favored their health and their babies. They also followed new hospital guidelines, such as using pumps and medical guidance on hand expression to provide breastmilk, though they were separated from their babies and were experiencing impaired bonding. Participants 4 and 10 articulated that suppressing negative emotions and self-management based on the guidelines relevant to COVID-19 helped reduce fears and motivated them to be adaptive.

*Theme 5: Acquiring resilience*

Finally, with the fear and separation being resolved with support and coping, mothers developed resilience unique from other outcomes from their challenges in previous perinatal experiences, as articulated by P6. Once reunited, women were able to reconnect with their babies, but for some women, the initial separation had already taken its toll. After separation, the reconnection that resolved impaired bonding was viewed as a remarkable experience, as reflected by P4. Women were now allowed to interact face-to-face, enhancing their postnatal bonding experience. However, fear and separation in the initial experience caused emotional and relational disruptions of the postnatal bonding, which may require additional interventions and periods to resolve.

### Point of integration

Fear and anxiety influenced mothers to avoid close contact, which explains the disruption in the postnatal mother–baby bonding process. Other factors include social distancing and clinical guidelines that disrupt early bonding opportunities, including skin-to-skin contact and breastfeeding initiation. Due to isolation guidelines, the family support system gap existed but was relieved by the healthcare providers. The preparedness and holistic support of the healthcare providers positively impact maternal well-being, which this study finds essential in a positive postnatal bonding experience during a pandemic. Several self-adaptive strategies helped mothers cope with the pandemic experience but could not seemingly resolve the essential effects of deeper mother–baby bonding. Hence, impaired bonding still exists, as revealed in the quantitative findings. The period of reconnection after separation somehow resolved impaired bonding and was viewed as a remarkable experience. However, the effects of interruption in emotional attachment during impaired bonding warrant further research.

## DISCUSSION

This study highlights three comprehensive findings about postnatal bonding during the COVID- 19 pandemic. Impaired bonding was experienced by mothers with COVID-19 due to fear and uncertainties, aside from evolving clinical guidelines on isolation. Although maternal age is known to influence psychological factors such as stress levels and fear of COVID-19 transmission often due to varying life experiences, responsibilities, and perceived vulnerability, our findings suggest that age is not significantly associated with the components of postnatal bonding scores. This may be because bonding is a complex, multifactorial process influenced more directly by immediate postpartum experiences, emotional support, delivery outcomes, and maternal–infant interactions rather than chronological age alone. While younger or older mothers may report different levels of stress or fear, these emotional states do not necessarily translate into measurable differences in bonding as captured by the Postpartum Bonding Questionnaire (PBQ). Additionally, bonding impairments may be more sensitive to situational and relational variables (e.g. quality of support, delivery experience, infant health) than to age. Both education level and mode of delivery were associated with impaired bonding. Social support from healthcare helped mothers cope and develop resilience amidst the difficulties during pandemics.

Several studies also explained that women experienced disruptive maternal–infant bonding during the COVID-19 pandemic period^15^. A previous study showed that the likelihood of impaired bonding during the pandemic was 2.56 times higher one month after delivery during the pandemic^19^. A Qatar study found anxiety and depression among pregnant and postnatal women due to social restrictions and family safety concerns^20^. Postpartum women separated from their newborns experience behavioral changes, leaving mothers with uncertainties about their condition^20,21^. Our study showed that narratives from mothers showed fear, overprotection, and evolving clinical guidelines illustrate impaired bonding. Fierstein et al.^22^ discussed that the mothers’ psychological distress due to disruptions in the mother–baby dyad and perinatal anxiety could likely lead to poor postnatal bonding. Our study also showed that mothers became less motivated to bond, and one woman stated self-reported depression. The rate of perinatal depression during the COVID-19 pandemic was increasing, which was attributed to isolation and other forms of restrictions leading to poor postnatal bonding^2^. In Saudi Arabia, lockdowns promoted postnatal bonding due to fewer visitors and easier breastfeeding^13^. In contrast, our study examined hospital and post-discharge experiences among women confirmed with COVID-19 infection. Aside from hospital restrictions, women were worried that they might harm their babies, which affects their well-being and decision-making towards postnatal bonding. During the pandemic, two predictors included mode of delivery and education level. Higher level of education is associated with high bonding scores, similar to previous studies during pre- and post-pandemic^23,24^. Assisted deliveries were also linked negatively with bonding, which in previous studies were only during pre-pandemic^25,26^. Our study and the citations discussed that these findings can be explained by negative emotions like fear, worries, pain, and discomforts. However, further studies can be done which compare mother’s experiences before, during and after the pandemic to get an overall perspective of the impact of COVID-19 to perinatal care.

Recent clinical studies suggested that breastmilk cannot cause vehicle transmission and contain protective immunoglobulins, which protect babies from the COVID-19 virus^27^. With the benefits of breastfeeding to both mothers and their babies, especially during immediate postnatal care, initiation of breastfeeding was recommended^28^. However, narratives from our study revealed that impaired bonding has directed less breastfeeding and skin-to-skin contact. Consistent with the study of Diaz et al.^29^, fear and anxiety among mothers have been associated with impaired bonding, which also lowers the chances of skin-to-skin contact and breastfeeding. In Qatar, the intent to not breastfeed has been associated with significant fear of COVID-19 mothers^15,16^. A previous study in UAE showed that infants separated from women diagnosed with COVID-19 have six-fold chances of not exclusively breastfeeding. Either suspected or confirmed COVID-19 cases, mothers experienced contradictions to either initiate or continue breastfeeding, which leads to infant separation^30^. The feeding disruptions were evident during the pandemic, especially during the first Phase, due to insufficient data and uncertainties^31^, and the new protocols like distancing and face masks^32^. Our study showed that mothers held positive views about breastfeeding, recognizing its logical importance as they accessed information from diverse sources. The World Health Organization recommends parental education on emotional needs for mother and baby and emphasizing skin-to-skin contact^26^. Our study revealed that addressing challenges with bonding and breastfeeding through education and support from healthcare professionals and online platforms, has improved coping and resilience. Online social media resources support mothers during lockdowns and isolation, helping them gain self-efficacy and bonding^13^. However, less support can lead to stress and anxiety, making breastfeeding unlikely during the pandemic^31^. Provision of support has been documented as a personal psychosocial variable that influences women to initiate and sustain breastfeeding^32^.

Our study showed that providing social support from staff, through their kindness and empathy, helped mothers cope better and build distinctive resilience. Social support has been proven effective in reducing maternal stress and promoting maternal–infant bonding^33^. Added to this is the insight of our study that self-caring plays a crucial part in coping with the challenges. Women showed resilience by accepting new ways, developing a positive response to their childbirth experience, and gaining lessons from the new experience. The success of maternal–infant bonding during the pandemic also relies on the mother’s confidence to care for their infants^16^. The gap in family support during hospitalization was relieved with family and friends. Though ‘face-to-face’ support can help recognize and solve practical issues^33^. Our study suggests that online support can somehow mend emotional concerns. Social support plays a key role as it is a protective factor for maternal mental health^30^. Thus, support is crucial in fostering attachment as the challenges compared to pre-pandemic were amplified as women transitioned to the postpartum period. The pandemic has increased maternal distress, which disrupts bonding and can alter infant behaviors in the future. This finding highlights the need to understand impaired bonding, its directional influences, and its overall relationship to the child’s well-being. In hindsight, it should have been paramount to give women information regarding the importance of breastfeeding and bonding, despite the risks posed by the pandemic. Thus, continued support is paramount during this unprecedented coronavirus outbreak as it does not have short- or long-term consequences to maternal and child health.

### Limitations

The online survey according to the calculated sample size and phone interviews of eleven eligible mothers who were conveniently recruited, limits the generalizability of the findings and increases the likelihood of selection bias and response bias. However, the study sequentially integrated both quantitative and qualitative findings, which offset the limitations of the survey and increased the credibility and dependability of the study findings. The validity of the Arabic-derived tool was limited to content validity and reliability.

### Implications

Based on the findings and insights of the present study, there are implications in practice, education, policy, and future research. In practice, provision of social support is found crucial for women’s adaptability to the perinatal care method, especially with new approaches like during the pandemic. Raising awareness through information dissemination in the community and inpatients are essential as the pandemic significantly altered postnatal care. Education about new hygiene measures, breastfeeding, visiting policies, and infection control procedures, has favorable outcomes to the mother’s well-being and engagement capacity. The findings of this study can help stakeholders and policy makers to develop perinatal wellness programs, implementing mental health assessment screening tools for early identification and therefore improve mother–infant interaction and maternal wellbeing. In research, further studies can be done like the long-term impact of impaired bonding during COVID-19 to both mothers and babies. Another potential direction for research includes interventions and clinical guidelines that either ensure positive perinatal outcomes or improved postnatal bonding during crisis like pandemic. If the study will be replicated in other countries, it is recommended that the specific period or wave relevant to the mother’s experience should be ascertained. Thus, in practice, education, and research, healthcare providers, particularly midwives, play a crucial role in promoting positive experiences for mothers during puerperium especially in crisis situations. Providing of holistic support, encouraging and reassuring women to bond with their newborns should be the core approach during crises. The pandemic highlighted the importance of healthcare professionals working together to navigate uncertainties and ensure efficient guidelines.

## CONCLUSIONS

Considering the changes in the delivery and postpartum period conditions due to the pandemic, it is plausible that these have negatively impacted maternal–infant bonding. With the PBQ score of >13, the study indicated that mother–infant bonding was present during COVID-19 in the selected facilities. The impaired bonding finding was also substantiated by the participants narratives in Themes 1 and 2 which highlight fear and separation. Integrating the findings provides a clearer substantiation that fear, and anxiety coupled with in-hospital protocol during the initial phase of the pandemic, could have contributed to the postnatal bonding score indicative of impaired bonding. The narratives further supported the identified findings and revealed the difficulties that women experienced in the perinatal period. These feelings were somehow mitigated with healthcare professional and social support.

## Data Availability

The data supporting this research cannot be made available for privacy or other reasons. Approval from the Review Board of the Hamad Medical Corporation is needed prior to data sharing.
